# Supplementary material for: Hippocampal connectivity patterns echo macroscale cortical evolution in the primate brain
Source: Nat Commun. 2024 Jul 16;15:5963. doi: 10.1038/s41467-024-49823-8 (PMC11252401; doi:10.1038/s41467-024-49823-8)
Supplement: Supplementary file 1 — Supplementary Information [file 41467_2024_49823_MOESM1_ESM.pdf]

Supplemental Information to:

Hippocampal connectivity patterns echo macroscale cortical evolution in the primate brain

Nicole Eichert[1,*], Jordan DeKraker[2], Amy F.D. Howard[1], Istvan N. Huszar[1], Silei Zhu[1], Jérôme Sallet[1,3], Karla L. Miller[1], Rogier B. Mars[1,4], Saad Jbabdi[1] & Boris C. Bernhardt[2]

[1] Wellcome Centre for Integrative Neuroimaging, Centre for Functional MRI of the Brain (FMRIB), Nuffield Department of Clinical Neurosciences, John Radcliffe Hospital, University of Oxford, Oxford, United Kingdom

[2] Department of Neurology and Neurosurgery, Montreal Neurological Institute and Hospital, McGill University, Canada

[3] INSERM U1208 Stem Cell and Brain Research Institute, Univ Lyon, Bron, France

[4] Donders Institute for Brain, Cognition and Behaviour, Radboud University Nijmegen, Nijmegen, The Netherlands

* corresponding author

Lead Contact:
Dr Nicole Eichert
nicole.eichert@ndcn.ox.ac.uk

This file includes: Figures S1-S3

Additional supplementary files can be accessed online with the manuscript:

- Supplementary Video 1: Hippocampal anatomy
- Supporting Data (Figshare: https://doi.org/10.6084/m9.figshare.25540171)

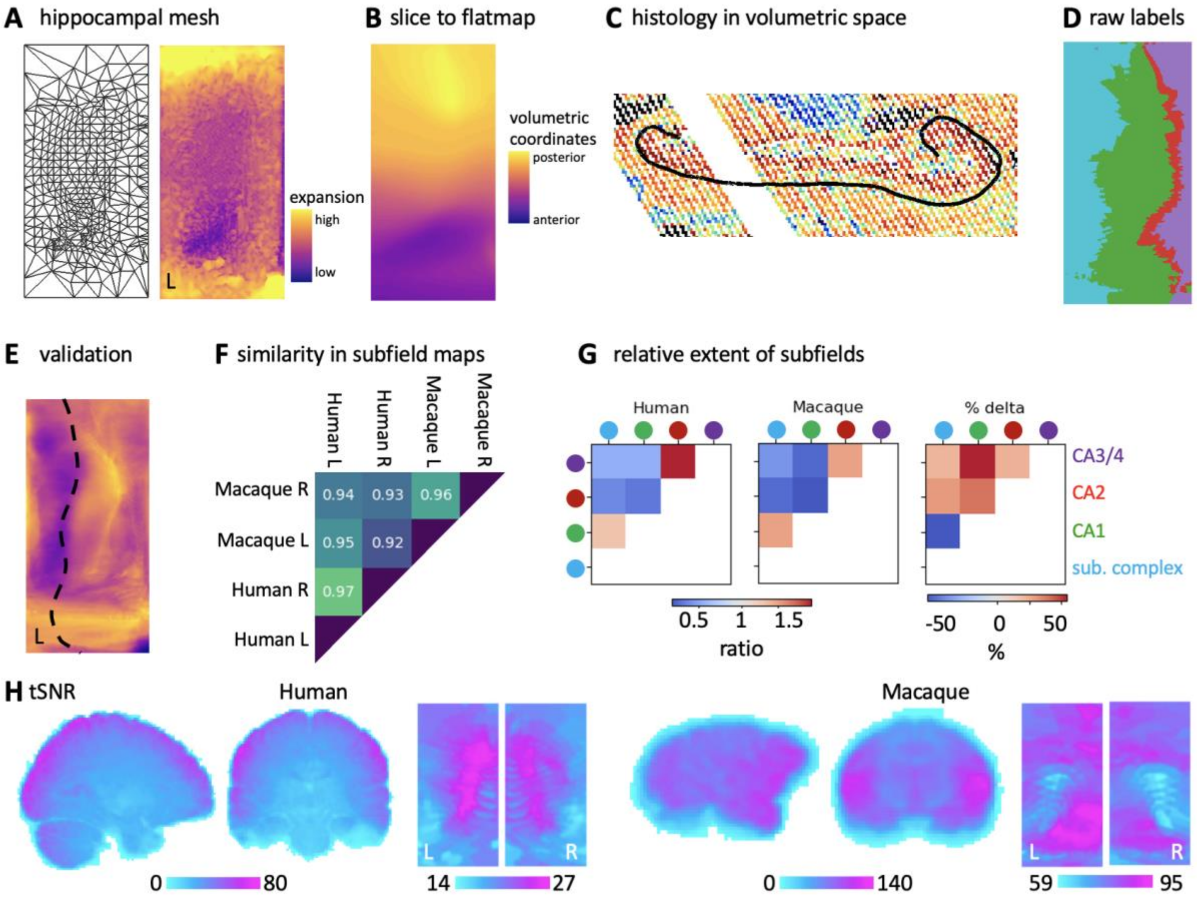

Figure S1. Related to Figure 1. A: Low-resolution hippocampal flatmap mesh and surface area of each vertex. B: The volumetric order of histology slices from posterior to anterior mapped to the hippocampal flatmap. C: Histology mapped to 3D volumetric space and hippocampal mid-thickness surface overlaid in black. D: Raw subfield labels mapped to the surface before correction on the surface. E: Average MGE map of two *ex-vivo* macaque brains. Dashed line: CA1-subicular complex boundary from BigMac. F: Cosine similarity of the hippocampal subfield maps across hemispheres and species. G: Extent of subfields relative to each other in human and macaque and percentage difference between human and macaque. H: Average tSNR maps (n = 10 individuals) of the rs-fMRI data for human and macaque shown in volumetric space and sampled to the hippocampal surface. Abbreviations: L = left, R = right, tSNR = temporal signal-to-noise ratio, sub. complex = subicular complex.

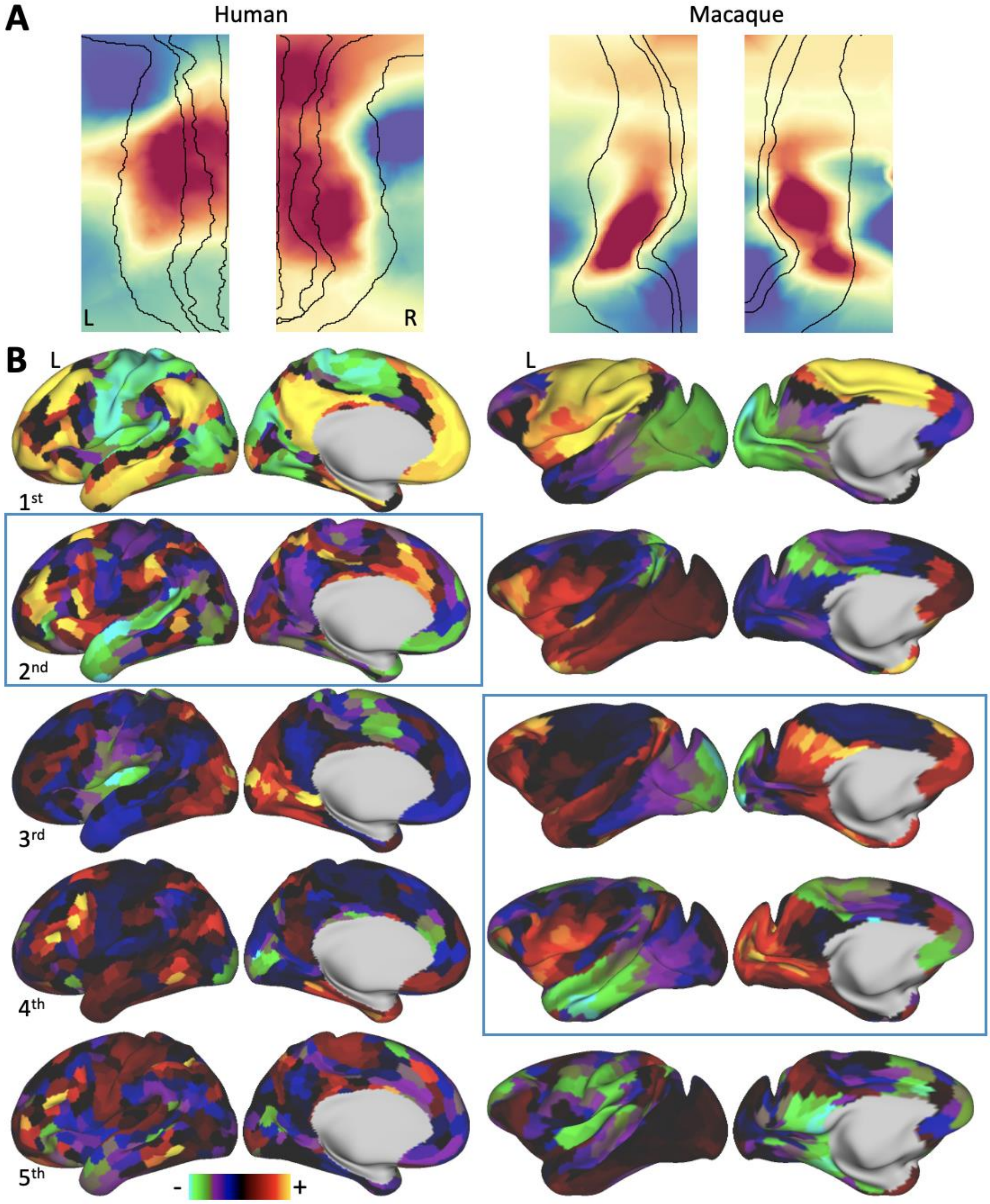

Figure S2. Related to Figure 2. A: Higher-order hippocampal gradients: 2nd human and 6th macaque hippocampal gradient displaying proximal-distal differentiation. The sign of the map is random. Subfield borders are indicated with black lines. B: Cortico-cortical gradients. The boxes highlight the gradients that match the joint cross-species gradient, i.e., the thresholded maps shown in Figure 2D.

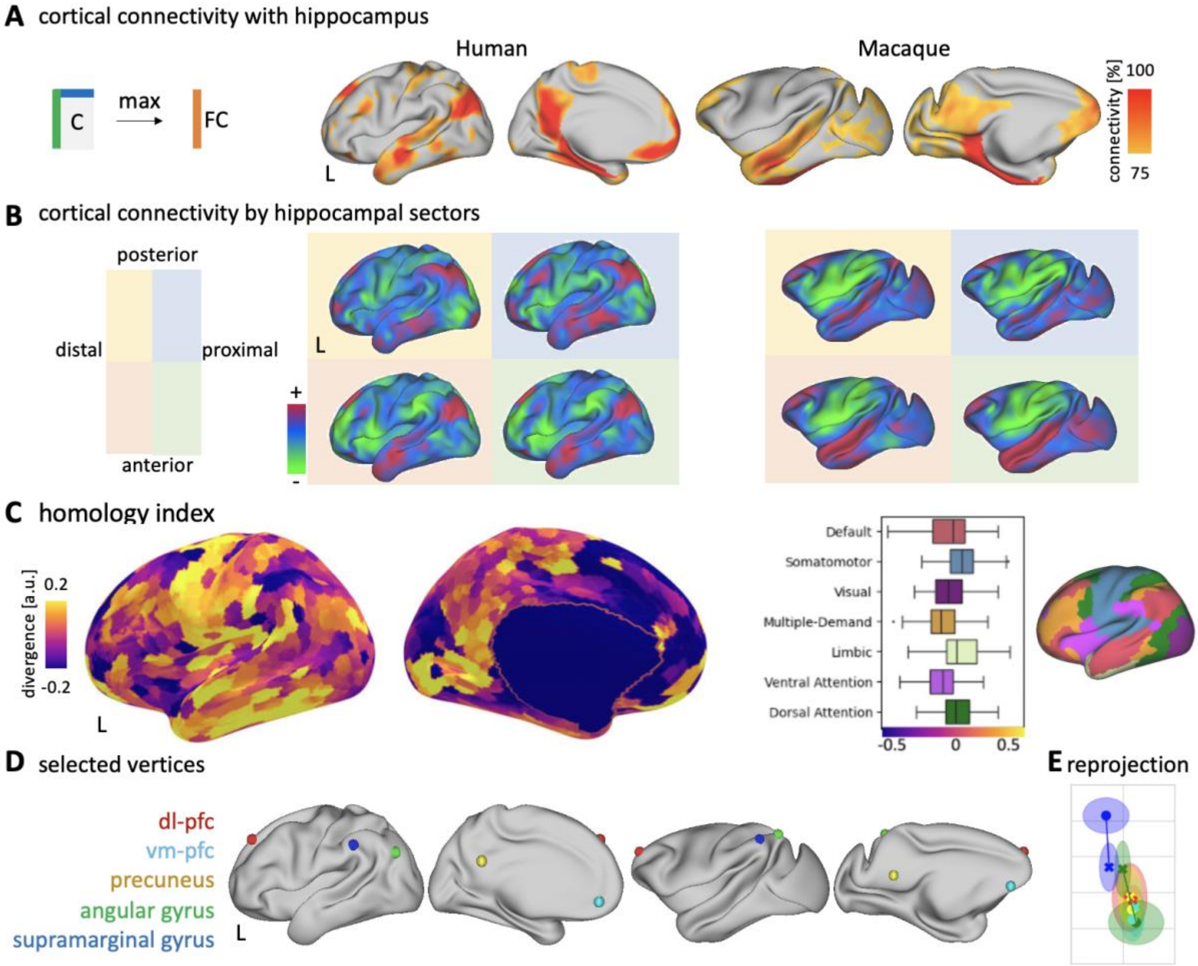

Figure S3. Related to Figure 3. A: Left - In each species, a functional connectivity map (FC) was obtained by computing the maximal connectivity of each cortical vertex from the cortico-hippocampal connectivity matrix (C). Right - Thresholded functional connectivity map (>75%, n = 10 individuals). B: Functional connectivity with average signal from four spatial sectors of the hippocampus. C: Homology index computed based on hippocampal connectivity profiles after applying a cross-species registration. Left - Whole-brain map. Right - Mean homology index per resting-state network[51]. The box plot indicates median (middle line), 25th, 75th percentile (box) and 5th and 95th percentile (whiskers) as well as outliers (single points). D: Selected vertices mapped in Figure 3B. E: Mean and standard deviation of the coordinates plotted in Figure 3B based on 10 individual subjects. Note the mean location in the 2D space is slightly different than in Figure 3B because the quantile-based thresholding of the axis in individuals differs from the threshold based on the group average. Abbreviations: C = connectivity matrix, FC = functional connectivity, L = left, a.u. = arbitrary unit.

Supplementary Video 1: Hippocampal anatomy. Hippocampal subfields manually labelled in a reference macaque brain (BigMac) are shown in several displays (left hemisphere only). Top Left - 3D rendering of the hippocampal subfields. A coronal and an oblique plane, orthogonal to the long-axis, are shown as transparent grey planes. Top Right - The unfolded 2D flatmap of subfields shown with the intersection of the coronal plane and the y-coordinate measured from the back of the brain. Bottom Left/Middle - The intersection of the MRI volume and subfields with both planes. Bottom Right - The histology slice approximately corresponding to the coronal section. The Dentate Gyrus (yellow) is included to provide orientation only.

Supporting Data (access via Figshare: https://doi.org/10.6084/m9.figshare.25540171).
Title: Hippocampal connectivity patterns echo macroscale cortical evolution in the primate brain.
Description: Supporting data for the manuscript Eichert et al. Nature Communications (2024). Related to Figure 1 in the main manuscript. Screenshots of Nissl-stained hippocampal histology slices from BigMac with manual hippocampal subfield labels. The y-coordinate indicates the approximate distance from the back of the brain. High-resolution histology slices and digital annotation files are provided via Oxford WIN's Digital Brain Bank (Dataset title: Hipmac project). For access to other data related to the manuscript, please refer to the Data Availability section of the main manuscript. For further questions, please contact nicole.eichert@ndcn.ox.ac.uk.
