# Supplementary material for: Hippocampal connectivity patterns echo macroscale cortical evolution in the primate brain
Source: Nat Commun. 2024 Jul 16;15:5963. doi: 10.1038/s41467-024-49823-8 (PMC11252401; doi:10.1038/s41467-024-49823-8)
Supplement: Supplementary file 3 — Description of Additional Supplementary Files [file 41467_2024_49823_MOESM3_ESM.pdf]

Description of Additional Supplementary Files

File Name: Supplementary Video 1
Description: Hippocampal anatomy. Hippocampal subfields manually labelled in a reference macaque brain (BigMac) are shown in several displays (left hemisphere only). Top Left - 3D rendering of the hippocampal subfields. A coronal and an oblique plane, orthogonal to the long-axis, are shown as transparent grey planes. Top Right - The unfolded 2D flatmap of subfields shown with the intersection of the coronal plane and the y-coordinate measured from the back of the brain. Bottom Left/Middle - The intersection of the MRI volume and subfields with both planes. Bottom Right - The histology slice approximately corresponding to the coronal section. The Dentate Gyrus (yellow) is included to provide orientation only.
