# Supplementary figures and images for: Hippocampal connectivity patterns echo macroscale cortical evolution in the primate brain
Source: Nat Commun. 2024 Jul 16;15:5963. doi: 10.1038/s41467-024-49823-8 (PMC11252401; doi:10.1038/s41467-024-49823-8)

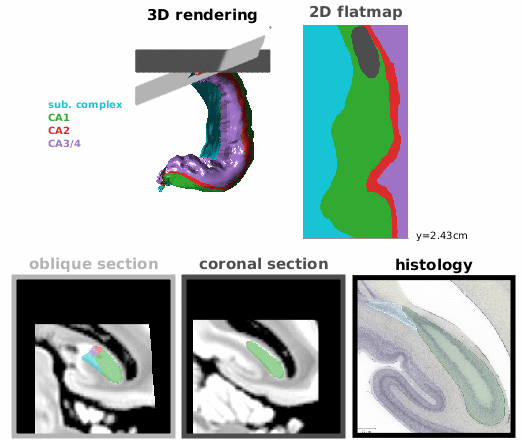

Supplement: Supplementary file 4 — Supplementary Video 1 [file 41467_2024_49823_MOESM4_ESM.gif]
