# Supplementary material for: Hippocampal connectivity patterns echo macroscale cortical evolution in the primate brain
Source: Nat Commun. 2024 Jul 16;15:5963. doi: 10.1038/s41467-024-49823-8 (PMC11252401; doi:10.1038/s41467-024-49823-8)
Supplement: Supplementary file 5 — Reporting Summary [file 41467_2024_49823_MOESM5_ESM.pdf]

nature portfolio

Corresponding author(s): Nicole Eichert

Last updated by author(s): May 30, 2024

Reporting Summary

Nature Portfolio wishes to improve the reproducibility of the work that we publish. This form provides structure for consistency and transparency in reporting. For further information on Nature Portfolio policies, see our Editorial Policies and the Editorial Policy Checklist.

Statistics

For all statistical analyses, confirm that the following items are present in the figure legend, table legend, main text, or Methods section.

n/a	Confirmed	
☐	☒	The exact sample size (*n*) for each experimental group/condition, given as a discrete number and unit of measurement
☒	☐	A statement on whether measurements were taken from distinct samples or whether the same sample was measured repeatedly
☐	☒	The statistical test(s) used AND whether they are one- or two-sided<br>*Only common tests should be described solely by name; describe more complex techniques in the Methods section.*
☒	☐	A description of all covariates tested
☐	☒	A description of any assumptions or corrections, such as tests of normality and adjustment for multiple comparisons
☐	☒	A full description of the statistical parameters including central tendency (e.g. means) or other basic estimates (e.g. regression coefficient) AND variation (e.g. standard deviation) or associated estimates of uncertainty (e.g. confidence intervals)
☐	☒	For null hypothesis testing, the test statistic (e.g. *F*, *t*, *r*) with confidence intervals, effect sizes, degrees of freedom and *P* value noted<br>*Give P values as exact values whenever suitable.*
☒	☐	For Bayesian analysis, information on the choice of priors and Markov chain Monte Carlo settings
☒	☐	For hierarchical and complex designs, identification of the appropriate level for tests and full reporting of outcomes
☐	☒	Estimates of effect sizes (e.g. Cohen's *d*, Pearson's *r*), indicating how they were calculated

Our web collection on statistics for biologists contains articles on many of the points above.

Software and code

Policy information about availability of computer code

Data collection	see Data section below.
Data analysis	No new software was developed. Custom wrapper code for existing software was written in Shell Script and Python 3.7 with standard Python packages to execute already published software, as detailed in the methods section. The custom wrapper code is available at https://git.fmrib.ox.ac.uk/neichert/project_hipmac. Specifically, we used the FMRIB Software Library (FSL v.6), HCP's Connectome Workbench v.1.2.3 (wb_command) and ANTS v.2.2.0 (http://stnava.github.io/ANTs/) to process MRI data. Cortical surfaces were generated with FreeSurfer v.7.2 (Fischl et al. Neuroimage, 2012). ITK-SNAP v.3.8 was used as interface to manually annotate the hippocampal segmentation. Hippocampal subfield maps were manually drawn in QuPath v.0.2.3 (Bankhead et al., Sci. Rep., 2017). Hippunfold v.0.3 (https://github.com/khanlab/hippunfold) was used to generate the hippocampal surface and the hippocampal flatmap. To map histology to MRI, we used existing registrations provided by the BigMac dataset, which were derived using TIRL v.3.1 (https://fsl.fmrib.ox.ac.uk/fsl/fslwiki/TIRL). Connectivity gradient embeddings were derived using Brainspace v.0.1.4 (https://github.com/MICA-MNI/BrainSpace).

For manuscripts utilizing custom algorithms or software that are central to the research but not yet described in published literature, software must be made available to editors and reviewers. We strongly encourage code deposition in a community repository (e.g. GitHub). See the Nature Portfolio guidelines for submitting code & software for further information.

Data

Policy information about availability of data

All manuscripts must include a data availability statement. This statement should provide the following information, where applicable:

- Accession codes, unique identifiers, or web links for publicly available datasets
- A description of any restrictions on data availability
- For clinical datasets or third party data, please ensure that the statement adheres to our policy

High resolution Nissl-stained histology data with digital annotation files, the CIVM template MRI tissue segmentation, as well as ex-vivo macaque MRI data have been deposited in the WIN's Digital Brain Bank platform (https://open.win.ox.ac.uk/DigitalBrainBank35; dataset title: Hipmac project). Macaque in-vivo MRI data have been made available via the Open Science Framework (DOI: 10.17605/OSF.IO/HKE98). Supporting Data with screenshots of the hippocampal sections with annotations are openly available on Figshare (https://doi.org/10.6084/m9.figshare.25540171) HCP data are publicly available at https://www.humanconnectome.org/. The CIVM macaque post-mortem template is publicly available at https://civmvoxport.vm.duke.edu/ (34). The BigMac data are available via the Digital Brain Bank platform (https://open.win.ox.ac.uk/DigitalBrainBank, 35; dataset title: The BigMac dataset). The human BigBrain hippocampal subfield map was accessed from a previous study (https://zenodo.org/record/6360647,105).

Research involving human participants, their data, or biological material

Policy information about studies with human participants or human data. See also policy information about sex, gender (identity/presentation), and sexual orientation and race, ethnicity and racism.

Reporting on sex and gender	For in-vivo MRI data, we accessed data from 10 HCP subjects (4 females) to match the sample size in the macaque. See more details for the HCP data release here: https://www.humanconnectome.org/study/hcp-young-adult/document/1200-subjects-data-release. The participant's gender was determined by self-report and is available via the HCP's open access data. Previously established human histological subfield mapping was based on a single post-mortem sample from a 65-year-old male donor. We did not consider sex-specific effects in this study, as our focus was to compare the human to the macaque and previous research suggested there were only negligible differences in hippocampal microstructure between sexes.
Reporting on race, ethnicity, or other socially relevant groupings	Race, ethnicity or other social variables were not accessed from the HCP database as they were not considered relevant for the comparison of the human to the macaque.
Population characteristics	The average age of the HCP participants was 30.4 ± 3.2 years. All participants were healthy as assessed via self-report.
Recruitment	Recruitment was performed by the HCP as described in the data release https://www.humanconnectome.org/study/hcp-young-adult/document/1200-subjects-data-release.
Ethics oversight	All HCP scanning protocols were approved by the local Institutional Review Board at Washington University in St. Louis. All subjects provided informed consent prior to participating in the study. The donor for the post-mortem BigBrain sample is not personally identifiable and gave written informed consent for the general use of post-mortem tissue used in this study for aims of research and education. The usage of the post-mortem material is covered by a vote of the ethics committee of the medical faculty of the Heinrich Heine University Düsseldorf (#4863).

Note that full information on the approval of the study protocol must also be provided in the manuscript.

Field-specific reporting

Please select the one below that is the best fit for your research. If you are not sure, read the appropriate sections before making your selection.

☒ Life sciences ☐ Behavioural & social sciences ☐ Ecological, evolutionary & environmental sciences

For a reference copy of the document with all sections, see nature.com/documents/nr-reporting-summary-flat.pdf

Life sciences study design

All studies must disclose on these points even when the disclosure is negative.

Sample size	The human sample size was determined to match that of the available macaque sample size. For histological mapping, a single human (BigBrain) and macaque (BigMac) were used as species representative. Given the whole-brain and multi-modal nature of these datasets they can serve as standard resources for anatomical characterizations. Comparable histological data at a similar sampling density and imaging resolution is currently not available as acquisition and curation are very time intensive. Two additional ex-vivo macaque brain scans were accessed to validate the microstructural map found in the single reference brain. The sample size for the functional analysis was similar to those in previous functional MRI studies in macaques, such as those listed below. This study capitalized on re-using existing open-access datasets to promote reproducibility and repeatability and to be in line with the principles of the 3Rs in animal research. Due to ethical guidelines within the UK, in-vivo non-human primate datasets can only be accessed if they comply with the UK National Centre for 3Rs (NC3Rs) guidelines.<br>Examples of previous comparative studies in macaques and humans with similar sample size:<br>- Mars, Rogier B., et al. Journal of Neuroscience 31.11 (2011): 4087-4100.

	- Giacometti, Camille, et al. Cerebral Cortex 32.18 (2022): 4050-4067.<br>- Roumazeilles, Lea, et al. Science advances 7.38 (2021): eabh2392.
Data exclusions	No subjects were excluded.
Replication	Two additional ex-vivo macaque brain scans were accessed to validate the microstructural map derived in the single macaque reference brain. Histological analysis in the macaque and the human were based on existing resources from a single brain in each species. Such resources are yet to be replicated elsewhere. This is due to the very time intensive nature of the data collection. Acquisition, pre-processing and curation of such multi-modal whole-brain datasets takes several years for a single brain.
Randomization	Randomization was not performed because participants were not placed into experimental groups.
Blinding	Blinding is not relevant to this study because participants were not placed into experimental groups.

Reporting for specific materials, systems and methods

We require information from authors about some types of materials, experimental systems and methods used in many studies. Here, indicate whether each material, system or method listed is relevant to your study. If you are not sure if a list item applies to your research, read the appropriate section before selecting a response.

Materials & experimental systems

n/a	Involved in the study	
☒	☐	Antibodies
☒	☐	Eukaryotic cell lines
☒	☐	Palaeontology and archaeology
☐	☒	Animals and other organisms
☒	☐	Clinical data
☒	☐	Dual use research of concern
☒	☐	Plants

Methods

n/a	Involved in the study	
☒	☐	ChIP-seq
☒	☐	Flow cytometry
☐	☒	MRI-based neuroimaging

Animals and other research organisms

Policy information about studies involving animals; ARRIVE guidelines recommended for reporting animal research, and Sex and Gender in Research

Laboratory animals	Macaca mulatta, 9 males, 1 female, age 7.2 ± 2.5 years, socially housed. Source: MRC, Centre for Macaques. NCBITaxon:9544.
Wild animals	No wild animals were used.
Reporting on sex	Data from 3 male macaque was used for the micostructural analaysis and data from 10 macaques (1 female) for the functional analysis. We did not consider sex-specific effects in this study, as our focus was to compare the human to the macaque and previous research suggested there were only limited differences in hippocampal microstructure between sexes. Accessing the same number of females and males in the macaque dataset was not possible, because only existing data were re-used in compliance with the NC3Rs guidelines.
Field-collected samples	No field-collected samples were used.
Ethics oversight	All experimental procedures in macaques were performed in compliance with the United Kingdom Animals (Scientific Procedures) Act of 1986. A Home Office (UK) Project License, obtained after review by the University of Oxford Animal Care and Ethical Review Committee, licensed all procedures. The housing and husbandry followed the guidelines of the European Directive (2010/63/EU) for the care and use of laboratory animals. The 3Rs principles compliance and assessment was conducted by the NC3Rs.

Note that full information on the approval of the study protocol must also be provided in the manuscript.

Magnetic resonance imaging

Experimental design

Design type	Ex-vivo post-mortem MRI and in-vivo resting-state functional MRI.
Design specifications	Humans: awake resting-state scan for approx. 14 min. Macaques: light anaesthesia during resting-state scan for approx. 1 h.
Behavioral performance measures	No behavioural performance was recorded.

Acquisition

Imaging type(s)	functional resting-state, structural, diffusion
Field strength	Human: 3T, Macaque: 7T
Sequence & imaging parameters	Human functional sequence: Gradient-echo EPI; TR: 720 ms, TE: 33.1 ms; flip angle: 52 deg; FOV: 208x180 mm (RO x PE); Matrix: 104x90 (RO x PE); Slice thickness: 2.0 mm; 72 slices; 2.0 mm isotropic voxels; Multiband factor: 8; Echo spacing: 0.58 ms; BW: 2290 Hz/Px. Macaque in-vivo functional: 1.5 mm3 spatial resolution, TR = 2280 ms, TE = 30 ms. Macaque in-vivo structural: magnetization-prepared rapid acquisition gradient echo (MPRAGE) sequence: 0.5mm3 isotropic. Macaque ex-vivo structural (multi gradient echo [MGE 3D]) sequence: TE/TR = 7.8/97.7 ms, flip angle = 30, 0.3 mm isotropic, resolution, FOV = 76.8 x 76.8 x 76.8 mm. Macaque ex-vivo diffusion-weighted spin echo multi-slice (DW-SEMS) sequence: TE/TR = 25.4 ms/10 s; FOV=76.8 x 76.8 x 76.8 mm; delta/Delta = 7/13 ms; time per gradient direction = 21.3 mins; b=4 ms/m2; 0.6 mm isotropic resolution; G = 32 G/cm;128 diffusion-weighted gradient directions; 8 volumes with negligible diffusion weighting.
Area of acquisition	Whole brain (both human and macaque)

Diffusion MRI ☒ Used ☐ Not used

Parameters: see above

Preprocessing

Preprocessing software	Both human and macaque MRI data was processed with the HCP-Pipeline (Human: https://github.com/Washington-University/HCPpipelines, Macaque: https://github.com/Washington-University/NHPPipelines). Specific tools: FMRIB Software Library (FSL 6.0): FLIRT and FNIRT for MRI-MRI registration, BET for brain extraction, FAST for wm/gm tissue segmentation, topup and eddy for susceptibility and eddy current distortion correction of in vivo diffusion MRI, melodic for ICA-based cleaning the resting-state fMRI. FreeSurfer for cortical surface reconstruction, HCP's connectome workbench (wb_command) for surface-based processing.
Normalization	Non-linear and linear volumetric and surface-based registrations were applied as part of the HCP-pipeline.
Normalization template	Human: MNI152 template, Macaque: Yerkes19 template.
Noise and artifact removal	Human: ICA-FIX, Macaque: manual ICA-based artifact removal as implemented in MELODIC
Volume censoring	not performed

Statistical modeling & inference

Model type and settings	(1) Multivariate analysis with feature selection via LASSO regression as implemented in scikit-learn (alpha = 0.1). Prior to the regression, data were transformed to a Gaussian distribution using scikit-learn's QuantileTransformer. To compute a goodness of fit, we used Ordinary Least Squares regression as implemented in the Python statsmodels package. (2) Kolmogorov-Smirnov test as implemented in Python's Scipy package with default settings.
Effect(s) tested	(1) Relationship between cortico-cortical gradient maps and the 1st joint cross-species gradient map. (2) We assessed the cross-species difference for each ROI in the 2D hippocampal space.

Specify type of analysis: ☒ Whole brain ☐ ROI-based ☐ Both

Statistic type for inference (See Eklund et al. 2016)	vertex-wise
Correction	(1) The significance of the correlations was determined using a spin test approach (1000 permutations) to control for spatial auto-correlations as implemented in BrainSpace. (2) The significance level was corrected for the number of comparisons performed.

Models & analysis

n/a	Involved in the study
☐	☒ Functional and/or effective connectivity
☒	☐ Graph analysis
☐	☒ Multivariate modeling or predictive analysis

Functional and/or effective connectivity	Functional connectivity was calculated based on Pearson's correlation.
Multivariate modeling and predictive analysis	Low-dimensional non-linear dimensionality reduction, i.e. diffusion map embedding, was performed as implemented in the GradientMaps function in Brainspace. Dual regression was performed by using two

orthogonal hippocampal maps as spatial regressors, fit to each individual's resting-state data to obtain beta maps of the second regression step.
